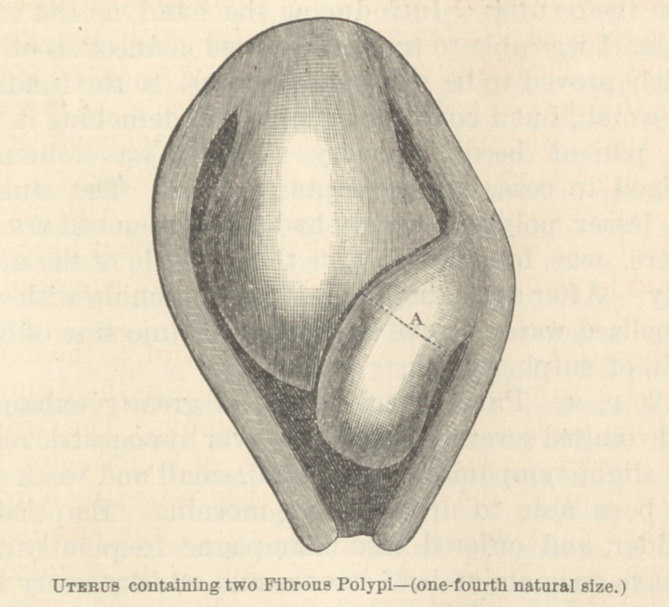# Remarks on Intra-Uterine Polypi, Etc.; with Three Cases

**Published:** 1876-03

**Authors:** A. Reeves Jackson

**Affiliations:** Surgeon-in-Chief of the Woman’s Hospital of the State of Illinois; Lecturer on Diseases of Women in Rush Medical College; Chicago


### SOME REMARKS ON INTRA-UTERINE POLYPI, WITH SPECIAL REFERENCE TO THEIR DIAGNOSIS AND SURGICAL TREATMENT; WITH THREE CASES.

Being the Report of the Gynecological Sub-Section of the Section on Obstetrics and Diseases of Women and Children.

(Read before the Chicago Society of Physicians and Surgeons, Nov. 22, 1875.)

By A. REEVES JACKSON, M.D.,

Surgeon-in-Chief of the Woman's Hospital of the State of Illinois; Lecturer on Diseases of Women in Rush Medical College.

(CONCLUDED.)

The following cases will serve to illustrate the principal points of diagnosis and treatment:

Case I. *Symptoms of Cancer—Removal of an Intra-Uterine Mucous Polypus—Rapid Recovery.*

Miss S., an unmarried woman 39 years of age, gave the following history: Three years ago she first noticed a leucorrhœal discharge of a yellowish glairy character. After the lapse of a few months it became thinner, more profuse, and sometimes had an offensive odor. At the same time her menstrual periods were increased in duration, although the quantity of the discharge was not notably more profuse. These symptoms were attributed to what the patient regarded as the approaching change of life, and she sought no advice. They became more and more severe, however, and during the past year the menstrual flow had been very abundant, the leucorrhœa watery in character, usually tinged with blood, and exceedingly offensive. It was so irritating as to produce an intolerable pruritus vulvæ. She had become greatly emaciated, her appetite was impaired and bowels constipated.

Here was a grave history, and I feared that the case was one of malignant disease. An examination revealed a short vagina with relaxed walls, and a soft, smooth, yielding cervix, without tenderness. The os was patulous, but not sufficiently to admit the point of the finger. The body of the uterus was not enlarged, and was not painful under pressure by conjoined manipulation. The sound, which was readily passed to the fundus at normal depth, yielded no further information, and its withdrawal was followed by a rather copious discharge of blood.

On the following day a sponge tent, of medium size, was introduced, and, although it seemed to fill the cervical canal, I succeeded in passing by its side three laminaria tents, each as large as a crow-quill. At the end of twenty-four hours these were removed, and I was able to introduce the index finger a short distance beyond the os internum which was amply dilated. About three-fourths of an inch beyond the external os the finger came in contact with a round, smooth, soft polypus the size of a gooseberry, attached by a short pedicle to the posterior wall of the cervix, just below the internal os. A forceps, such as is used for the removal of nasal polypi, was passed into the cervical canal, and, guided by the finger, its blades were made to grasp the peduncle. The instrument being rotated two or three times on its axis, the growth was detached and brought away. Fuming nitric acid was applied to the whole interior of the cervix and the patient put in bed.

The symptoms ceased at once, and the patient, now forty-two years of age, menstruates in all respects normally—or, at least, did so a few months ago.

Case II. *Menorrhagia, with great Enlargement of the Womb—Two large Fibrous Polypi—Removal of one by the Ecraseur, and the other partially thrown off Spontaneously—Death from Metro-Peritonitis.*

In March, 1874, I was invited, through the courtesy of Dr. F. A. Emmons, to see Mrs. W., aged 45 years, residing at Hinsdale, in this State. She was the mother of three children, the youngest of whom was eleven years old. Two years before, the catamenial flow became greatly increased, and was so excessive in quantity and its duration so prolonged, that she was frequently obliged to lie in bed ten days at a time. The discharge was accompanied by pain of an expulsive character, and the frequent escape of large clots from the genitals. She had constant leucorrhœa, yellowish and glairy, during the intermenstrual periods. She became rapidly anemic, and suffered from loss of appetite and sleep.

The uterus was found much enlarged, its fundus being felt two inches below the umbilicus, and somewhat higher on the right side than on the left. A metallic sound was introduced to the depth of six and a half inches, and one of a flexible material reached three-fourths of an inch farther. It was evident that the womb was occupied by a large tumor. Its character and place of attachment could only be ascertained by dilatation of the cervix. I advised that this be done by the use of sponge tents, and that trial be made of the hypodermic use of ergot. The sponge tent was not, I believe, successfully employed, and the differential diagnosis consequently not made. I learned, however, through Dr. Emmons, that the patient experienced much benefit from the ergot; pain and hæmorrhage were lessened, appetite and sleep were improved, and a considerable degree of strength returned. The remedy was continued, I think, seven or eight months. It was then omitted, and the symptoms soon reappeared with almost their former severity. I then saw the patient again in consultation with Dr. Emmons, and found the condition of the parts apparently the same as at the first examination, and advised an operation for the removal of the growth, provided removal were found possible with no more than ordinary risk. A fortnight later the patient came to the city for this purpose and was placed under my care.

On December 11th, with the assistance of Prof. De

Laskie Miller, I introduced one sponge tent and five laminaria tents. On the following day, again assisted by Prof. Miller, the tents were removed. Finding the internal os insufficiently dilated, we completed the dilatation by means of Molesworth's Dilator, with the greatest facility. Then, introducing the finger as far as possible into the cervix, a smooth, roundish body, as large as a hen's egg, was distinctly felt. Its attachment seemed high up, and was beyond the reach of the finger. It was decided at once to attempt its removal. Although the patient was admonished that the subsequent procedures would be painful, and perhaps prolonged, she resolutely refused to take any anæsthetic.

*Operation.*—The patient being drawn to the extreme end of a firm lounge, she was placed in the lithotomy position, and her limbs held by assistants. The perineum was drawn downward by means of a retractor. The anterior lip of the os uteri was then seized by a double-toothed vulsellum, by which the part was steadied and drawn downward. A larger and stronger vulsellum, guided by a finger previously introduced, was then passed into the cervix, and its hooked blades expanded over the polypus, which was grasped by them at a point as high up as the vulsellum could be passed—somewhat more than an inch and a half above its most depending part. The polypus was now drawn slowly and steadily down toward the vulva, this movement being aided by firm pressure over the hypogastrium. We finally succeeded in bringing the growth fairly into view just within the vulva, but still partially enclosed by the os uteri. The loop of a wire écraseur was now passed over the handles of the vulsellum and pushed by the finger beyond its toothed extremity and on to the polypus. It was found impossible to pass the loop very high up on the pedicle in consequence of some obstruction which was encountered. Having adjusted the loop as accurately and at as high a point as possible (see figure, point A), it was slowly tightened. In about five minutes a portion, the size of a hen's egg, was cut through and brought away. The patient's strength was now well-nigh exhausted and stimulants were freely administered.

UTERUS containing two Fibrous Polypi—(one-fourth natural size.)

She rallied slowly but perfectly, and during the three following days was quite comfortable, being free from pain, and sleeping and eating tolerably well. There was, however, a constant and copious discharge of a bloody serous fluid so offensive in odor that frequent vaginal injections of carbolized water were necessary.

Dec. 16th. Patient had an attack of very severe expulsive pain, which was quieted by a full dose of morphia. On the following day Prof. Miller visited the patient in my stead. After his visit, expulsive pains again occurred and lasted about an hour. They were accompanied by nausea and chills.

Dec. 18th, 10 A. M. Passed a restless night, but without much pain. Introducing a finger to the vagina, I found a large, soft, decomposing mass as large as an orange, which I could trace into the cervical canal, now greatly relaxed and expanded. Without much difficulty I

passed the hand into the vagina, and then firmly holding the lower portion of the mass with a vulsellum, I managed to tear away, piece-meal, so much of it as had protruded from the os uteri. Introducing the hand to the vagina again, I was able to trace the broad connection of this, which proved to be a second polypus, to the fundus of the womb, but I could not succeed in detaching it, and, the patient becoming very weak, I was reluctantly obliged to cease my attempts to do so. The stump of the lesser polypus, which had been removed six days before, was found to be on the left side of the uterine body. After syringing the vagina thoroughly with warm carbolized water, I gave a hypodermic injection of half a grain of sulph. morph.

8.30 P. M. Patient very pale, and greatly exhausted. Had vomited several times. Pain over hypogastric region, and slight tympanites; pulse, 140, small and weak; had not been able to urinate since morning. Emptied the bladder, and ordered iced champagne frequently; also to have enemata of beef essence and whisky every three hours.

Dec. 19th, 10 A. M. Patient pale and haggard; slept very little during the night; pulse 140, very feeble; tongue dry and dark; abdomen tense and tender. Introduced catheter; to continue beef and whisky enemata.

7 P. M. Prof. Miller saw the patient with me. She seemed much the same as in the morning, although the tongue was moist, and she expressed herself as feeling better. An examination discovered the remainder of the polypus widely distending the os uteri. The patient's exhaustion was so great, however, that any attempt at removal was deemed inadvisable.

This was the last time I saw the patient alive. I was confined to bed by illness the two following days, and Prof. Miller kindly visited her for me. She continued to sink, and expired at half past twelve on the 21st.

CASE III. *Intra-Uterine Polypi Complicated with Fissure of the Anus—Removal of a Fibrous Polypus and Several Cystic Polypi.*

Mrs. B., aged 35 years, consulted me Nov. 12th. She had been married thirteen years; had an abortion at two and a half months in the first year of her marriage, and had not been pregnant since. Menstruation, which began at the age of fourteen, was always painful and rather profuse. Within the past few years the quantity of the discharge has been much greater than before, and now habitually lasts from eight to ten days. Has always since puberty had more or less leucorrhœa; this, too, has been more copious within the last two or three years. It is thick, glairy, sometimes tinged with blood, and, usually, offensive in odor. She has been under the care of a number of physicians, most of whom have treated her for "ulceration" or "displacement," some of them without making a vaginal examination. Her appetite is good, she sleeps well, and her general health seems not much impaired. Her bowels are open daily, but defecation is attended and followed by pain which lasts several hours.

On examination, I found the uterus rather low in the pelvis, retroverted and slightly flexed. The vaginal portion was enlarged and the os partially eroded but not patulous. The sound was readily passed to the fundus, and indicated a depth of two and three-fourths inches. Its passage caused no pain, but its withdrawal was followed by bleeding. On the anterior verge of the anus there was a small condylomatous growth which the patient had always regarded as a hæmorrhoid; extending inward from it was an angry-looking fissure about three-fourths of an inch long, and directly opposite was another of the same character but of smaller size.

Nov. 16th. The patient having been thoroughly purged the previous day, I introduced a laminaria tent to the cervix, and directed the patient to remain in bed and to use a diet exclusively of milk porridge.

Nov. 17th. The patient having been fully etherized by Dr. D. A. K. Steele, I removed the tent, and was able, by using some force, to pass the finger into the uterine cavity. The interior of the cervix was fairly studded with projecting masses, the largest not exceeding a pea in size. They were non-pedunculated, smooth and somewhat flattened by the pressure of the tent. They were evidently cystic polypi, and occupied the lower half of the cervical canal. Above these, attached to the left side of the cervix by a broad base, was a larger growth, about the size of a chestnut.

The hips of the patient, who was on her back, were brought beyond the end of the table, and the lower extremities supported by assistants. A vaginal retractor was introduced and the os uteri readily brought into view. Seizing the anterior lip with a vulsellum, the uterus was drawn down to the vulva and the retractor withdrawn. A curette was now passed into the cervical canal, and, by its aid, all the polypi were removed in a few minutes. Very little hæmorrhage attended the operation. The interior of the womb having been cleansed of blood, was thoroughly swabbed with strong nitric acid, and the cervix having been released, a ball of cotton wool, saturated with glycerine, was placed against the os and pushed up into the vagina. The fissured anus was treated by forcible dilatation of the sphincter.

The larger polypus was distinctly fibrous in character.

Nov. 20th. Thus far the condition of the patient has been quite satisfactory. Without the use of any anodyne she has slept well every night. Her bowels were opened naturally on the fourth day without any pain whatever. There is an offensive bloody discharge proceeding from the genitals, caused by the separation of the nitric acid slough. The after-treatment has consisted of rest in bed, a diet of milk porridge, and a vaginal injection of warm carbolized water, used three or four times a day.

The medical treatment of polypus does not properly come within the scope of this paper; but there is one medicine, to the efficacy of which I desire to bear testimony. I allude to ergot. Its use is especially indicated in cases where growths of fibrous character are passing from the condition of submucous tumor to that of polypus; and also in cases where polypi of any kind have come to press against, and do not quite pass through the os uteri. In these cases the ergot, by inducing firm contraction of the uterine walls, forces the growth downward; it becomes more quickly and decidedly pedunculated, and consequently more accessible to radical treatment. At the same time it is the most efficient agent under these circumstances in checking the hæmorrhage. Prof. Byford has informed me that it has been his custom for many years to rely chiefly upon this drug for the purposes mentioned.

---

DID HE RECEIVE HIS DIPLOMA FROM PHILADELPHIA? —The *Lyon Médical* reports the arrival and performances in that city of one "Doctor Bribosia, a graduate of a foreign university." He evidently belongs to that peripatetic class whose visitations should be viewed as a calamity with which the grasshopper plague is not to be compared.

Here are some of the achievements of another of these charlatans, described in *l'Année Médicale du Calvados:*

A virgin, fifteen years old and consumptive, was in despair in consequence of her amenorrhœa. She was "deflowered" by the application of a pessary, when an abundant flow of blood was induced, which was soon followed by death.

Another virgin, sixteen years old, was submitted to the same treatment for relief of leucorrhœa due to chlorosis.

A poor old man had a hernia, and had worn out his truss supplied by a physician. The charlatan applied an apparatus, and, in eight days, the patient died of gangrene of the scrotum.

## Figures and Tables

**Figure f1:**